# Ultrastructural Modifications in the Mitochondria of Hypoxia-Adapted *Drosophila melanogaster*

**DOI:** 10.1371/journal.pone.0045344

**Published:** 2012-09-19

**Authors:** Guy Perkins, Yu-hsin Hsiao, Songyue Yin, Jonathan Tjong, My T. Tran, Jenna Lau, Jin Xue, Siqi Liu, Mark H. Ellisman, Dan Zhou

**Affiliations:** 1 National Center for Microscopy and Imaging Research, University of California San Diego, La Jolla, California, United States of America; 2 Department of Pediatrics, University of California San Diego, La Jolla, California, United States of America; 3 Beijing Institute of Genomics, Chinese Academy of Sciences, Beijing, P.R. China; Instituto de Química - Universidade de São Paulo, Brazil

## Abstract

Chronic hypoxia (CH) occurs under certain physiological or pathological conditions, including in people who reside at high altitude or suffer chronic cardiovascular or pulmonary diseases. As mitochondria are the predominant oxygen-consuming organelles to generate ATP through oxidative phosphorylation in cells, their responses, through structural or molecular modifications, to limited oxygen supply play an important role in the overall functional adaptation to hypoxia. Here, we report the adaptive mitochondrial ultrastructural modifications and the functional impacts in a recently generated hypoxia-adapted *Drosophila melanogaster* strain that survives severe, otherwise lethal, hypoxic conditions. Using electron tomography, we discovered increased mitochondrial volume density and cristae abundance, yet also cristae fragmentation and a unique honeycomb-like structure in the mitochondria of hypoxia-adapted flies. The homeostatic levels of adenylate and energy charge were similar between hypoxia-adapted and naïve control flies and the hypoxia-adapted flies remained active under severe hypoxia as quantified by negative geotaxis behavior. The equilibrium ATP level was lower in hypoxia-adapted flies than those of the naïve controls tested under severe hypoxia that inhibited the motion of control flies. Our results suggest that the structural rearrangement in the mitochondria of hypoxia-adapted flies may be an important adaptive mechanism that plays a critical role in preserving adenylate homeostasis and metabolism as well as muscle function under chronic hypoxic conditions.

## Introduction

Chronic hypoxia (CH) is characterized by a constant, long-lasting reduction in oxygen supply to tissues and cells. Such oxygen reduction occurs under certain physiological or pathological conditions, including in people who reside at high altitude or suffer chronic pulmonary diseases. One of the responses in organisms to CH is to decrease energy generation through marked suppression of adenosine 5′-triphosphate (ATP) demand and supply pathways [1]. As mitochondria are the predominant organelles for oxygen utilization in the cell, their regulatory response to altered oxygen supply is proposed to play an important role in the overall functional adaptation to hypoxia. Indeed, previous studies have shown that mitochondrial metabolism is involved in CH adaptation via energy regulation, generation of reactive oxygen species (ROS), and apoptosis [2], [3]. It is well established that mitochondria are remarkably plastic organelles that actively change their shape. Hypoxia-induced alterations of mitochondrial metabolism are thought to evoke changes in the mechanisms regulating mitochondrial structural dynamics. Such hypoxia-induced changes are widely considered as important pathogenic factors that contribute to hypoxic/ischemic cell injury in various organs including brain, heart, kidney and liver [4], [5], [6], [7], [8]. Although hypoxia-induced alterations on the dynamics of mitochondrial network formation in the cell have been reported [9], to date, the detailed three-dimensional (3D) structural modifications within mitochondrion have not been investigated. As functional modifications in mitochondria may play important roles in protecting cells from hypoxic injury [10], [11], it is important to determine the hypoxia-induced changes in mitochondrial ultrastructure and the impact on mitochondrial function.

As a well-studied organism, *Drosophila melanogaster* offers many useful features that are convenient for dissecting the mechanisms underlying different kinds of human diseases. These advantages include high degree of conservation in fundamental biological pathways between *Drosophila* and humans [12], a shorter generation time and life span, a large number of progeny, the availability of many well characterized genetic tools to manipulate gene function, and relatively well-known anatomy and phenotypes [13], [14]. To date, various *Drosophila* lines have been successfully generated to analyze the function of different human disease genes including those responsible for developmental and neurological disorders, cancer, cardiovascular disease, and metabolic and storage diseases [15], [16], [17], [18], [19]. The extensive conservation of mitochondrial structure, composition, and function across species also offers a unique opportunity to expand our understanding of human mitochondrial biology and disease in fruit fly [20], [21], [22].

In order to dissect the mechanisms underlying hypoxia tolerance, we recently generated a hypoxia-tolerant *Drosophila melanogaster* strain through laboratory selection that survives severe, otherwise lethal, hypoxic conditions [23]. Using this unique animal line, we have identified genes and pathways that regulate this remarkable hypoxia tolerance phenotype which include the role of transcription suppressor hairy and the activation of the Notch pathway [24], [25]. Furthermore, a synchronized global suppression of metabolic genes, including those encoding mitochondrial proteins, was discovered in the hypoxia-adapted (HA) flies [25]. Interestingly, metabolic profiles in the HA and normoxic control (NC) flies following acute hypoxia exposure revealed that HA flies had a higher efficiency of ATP production, lowered pyruvate carboxylase flux and greater usage of Complex I over Complex II respiration [26], demonstrating that mitochondrial adaptive adjustment, through the modifications of structure and enzyme activities, is one of the key factors regulating survival under chronic long-lasting hypoxic condition. In current study, we used HA thoracic muscle as a system to study the alterations in mitochondrial ultrastructure and the functional impact in this hypoxia-adapted organism.

## Results

### Muscle Structure and Mitochondrial Volume Density in HA Thoracic Muscle

**Figure 1:**
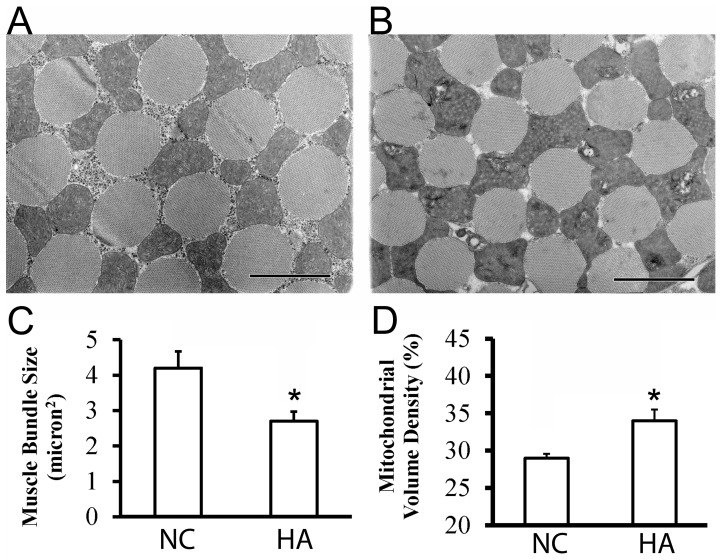
Muscle structure and mitochondrial volume density in HA thoracic muscle. (A) and (B): Typical electron micrographs of thoracic muscle from control (NC) and hypoxia-selected flies (HA) (scale  = 1 µm). No significant modifications on myofiber architecture were determined in the HA muscle as compared to that of NC control. However, a reduced myofiber size (C) (n = 69, *p<0.01) and an increased mitochondrial volume density (volume fraction) (D) (n = 11, *p<0.01) were found in the thoracic muscle of HA flies.

Typical electron micrographs of thoracic muscle from control (NC) and hypoxia-selected flies (HA) are shown in Figure 1A and 1B. The myofiber architecture was unaltered in the HA muscle compared to the NC muscle. However, the myofiber size from HA flies (2.7±0.3 µm^2^) was smaller than those of NCs (4.2±0.7 µm^2^) (p<0.01) (Figure 1C). In contrast, an increased mitochondrial volume density (volume fraction) was observed in the HA flies (34±1.5%) as compared to NC controls (29±0.6%) (p<0.01) (Figure 1D). The smaller myofibers in HA partially produced empty space between these fibers.

### Altered Mitochondrial Ultrastructure in HA Thoracic Muscle

**Figure 2:**
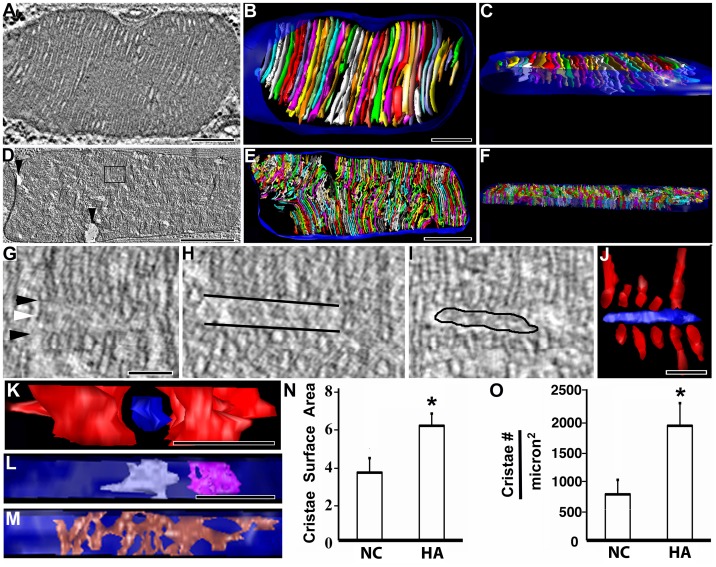
Ultrastructural modifications in the mitochondria of HA flies. (A) Slice through a tomographic volume of a normoxic mitochondrion found in thoracic muscle sliced longitudinally. Normal morphology is observed, including stacks of ordered cristae with light intracristal space and darker matrix. Scale  = 200 nm. (B) Segmented volume of the mitochondrial membrane structure. The cristae (various colors) were all found to be lamellar in form, as expected. This mitochondrial volume contained 42 cristae. Scale  = 200 nm. (C) Oblique view of the mitochondrion viewed partially through the outer membrane (blue) made translucent to observe the cristae arrangement. Same scale as B. (D) Slice through a tomographic volume of hypoxic thoracic muscle. This large mitochondrion displays a few regions devoid of cristae (arrowheads) and more notably, small regions where the cristae have been fractured (boxed example and shown expanded in G-I). Scale  = 1000 nm. (E) Top view of the membrane segmentation of the volume. This mitochondrial volume contained 641 cristae. Scale  = 1000 nm. (F) Oblique view of the mitochondrion viewed partially through the outer membrane to observe the cristae motif. Same scale as E. (G) Expanded view of the boxed region in D. This area and others in (H) and (I) from different slices of the volume show views of rod-like dense cores between fractured cristae. The edge of the rod is the fracture curve of the cristae (black arrowheads) where the cristae in a localized region fractured almost uniformly (black lines in H) and then the ends annealed, i.e., the cristae membrane closed around the break. The dense core (white arrowhead) appears to be the degenerated cristae (black outline in I) that are separated by a white (translucent) band from the annealed cristae membrane curve. Scale  = 100 nm. (J) Top view of the segmented region of I showing the dense core (blue) surrounded by the cristae fragments (red). Scale  = 100 nm. (K) Side view perpendicular to the view in J, down the axis of the dense core appearing almost as an avenue with the cristae as spaced sentinels. Scale  = 100 nm. (L) Examples of two lamellar cristae in the hypoxic mitochondrion of panel D. These cristae were not as extensive as those in the control and often exhibited fenestrations (arrowhead). A greater percentage of cristae were similar to the highly branched and severely fenestrated crista shown in (M) (* p<0.01). Scale  = 500 nm. (N) The cristae surface area, normalized to the mitochondrial outer membrane surface area, was significantly greater in the hypoxic thoracic muscle as was the cristae number, normalized to the mitochondrial cross-sectional area (O) (* p<0.01).

Electron tomography was used to characterize in 3D the membrane architecture of NC and HA thoracic muscle mitochondria (movie S1 and S2). The NC mitochondria appeared typical for thoracic muscle with densely packed lamellar cristae (Figure 2A–2B). We found that HA mitochondria showed localized swollen regions devoid of cristae (Figure 2D–2F) and more notably, small regions where the cristae have been sliced or fractured almost uniformly in cross-section as if a microscopic knife had sliced perpendicular to the long axis of 6 or more adjacent cristae and then the ends annealed, i.e., the cristae membrane closed around the break (Figure 2G–2I). Rod-like dense cores were often observed between the ends of the fractured cristae running perpendicular to the cristae membranes. The dense core appears to be almost as an avenue with the cristae as spaced sentinels (Figure 2J, 2K); they are likely the residue from cristae fragmentation and are separated by a light (translucent) band from the annealed cristae membranes. We also noted that the lamellar cristae in hypoxic mitochondria were not as extensive (large) as those in the control and often exhibited fenestrations (Figure 2L). A greater percentage of cristae in the HA thoracic muscle mitochondria were similar to the highly branched and severely fenestrated crista shown in Figure 2 M than those in the NC mitochondria. The cristae membrane surface area, normalized to the mitochondrial outer membrane surface area, was significantly greater in the hypoxic thoracic muscle (Figure 2N) as was the cristae number, normalized to the mitochondrial cross-sectional area (Figure 2O) compared with the NC mitochondria. The increase in cristae number reflects the cristae fragmentation noted and the increase in cristae membrane may be an adaptation to compensate for this fragmentation. The cristae width was found to differ between the longer cristae and the fragmented (and annealed) cristae in the HA mitochondria. The fragmented cristae in the HA mitochondria had a mean width of 31±0.5 nm (mean ± SEM, n = 100), whereas the longer cristae in the same mitochondria had a mean width of 23±0.3 nm (n = 100), a difference highly statistically significant (p<0.001), and nearly identical to the width of 24±0.4 nm (n = 100) measured for the NC mitochondria.

**Figure 3:**
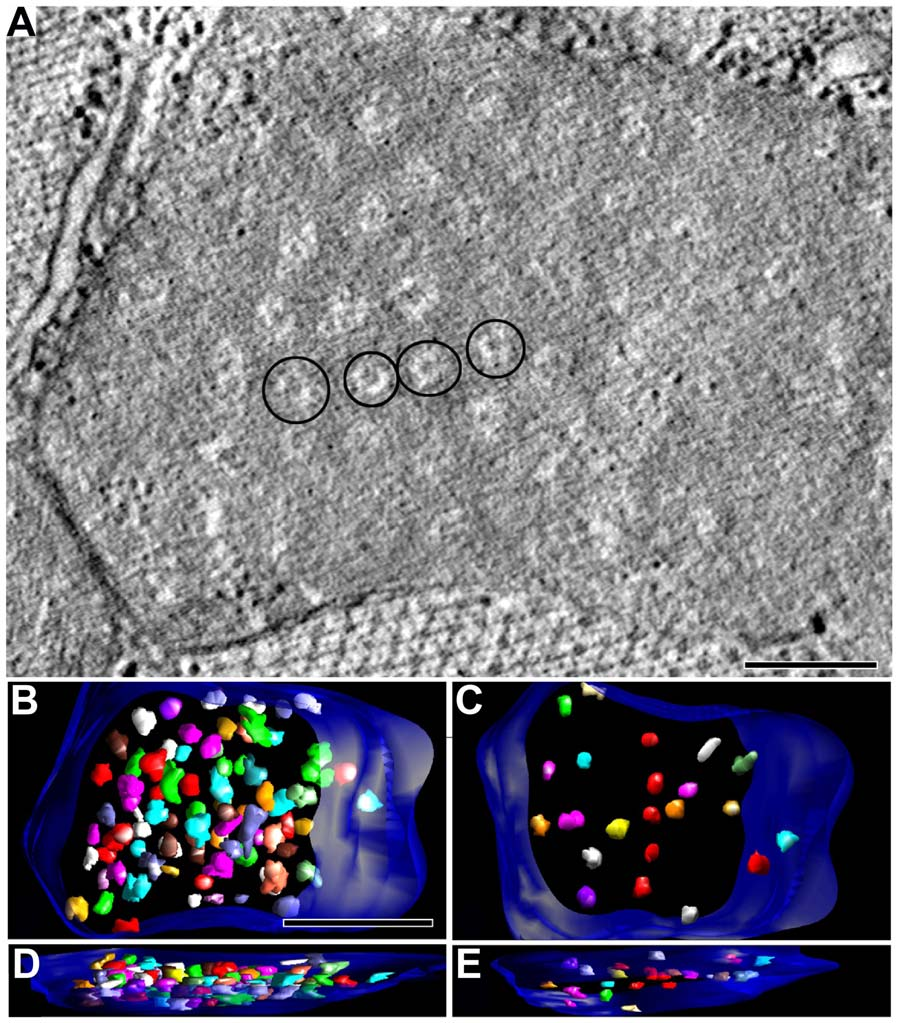
Honeycomb-like structures in the mitochondria of HA flies. (A) Slice through another tomographic reconstruction of a hypoxic mitochondrion in thoracic muscle oriented in cross-section (transverse) to the myofibrils. A honeycomb-like pattern of light, roughly circular regions with a dense center (4 circled) was found. Scale  = 200 nm. (B–E) The segmented honeycomb-like pattern (various colors) shows the spatial distribution and 3D extent of these structures. The full complement of rod-like structures having a dense core that is also rod-shaped, yet narrower is shown in B (top view) and C (oblique view). This mitochondrial volume contained 84 rods in the honeycomb-like structures. A subset of rods (23 total) is shown in D (top) and E (oblique) to note that these rods do not extend completely through the volume (height) of the mitochondrion. Scale  = 500 nm.

A honeycomb-like pattern of light, roughly circular regions with a dense center was commonly observed in HA muscle mitochondria when the tomographic reconstruction was sliced in cross-section to the myofibrils (Figure 3A). The dense center is the cross section of the rod-like core. The segmented honeycomb-like pattern shows the spatial distribution and three-dimensional (3D) extent of these structures (Figure 3B–3E). These rods do not extend completely through the volume of the mitochondria, consistent with the views of their limited extent shown in Figure 2. Measurements were made from electron micrographs to determine the prevalence of this honeycomb-like pattern. Only 1% of the NC mitochondria showed the honeycomb-like pattern (n = 175), whereas 49% of the HA mitochondria had this pattern (n = 380). Other HA mitochondria appeared normal.

### Equilibrium Adenylate Contents and Hypoxia-induced Changes in ATP Level in the Thoracic Muscle of NC and HA Flies

**Figure 4:**
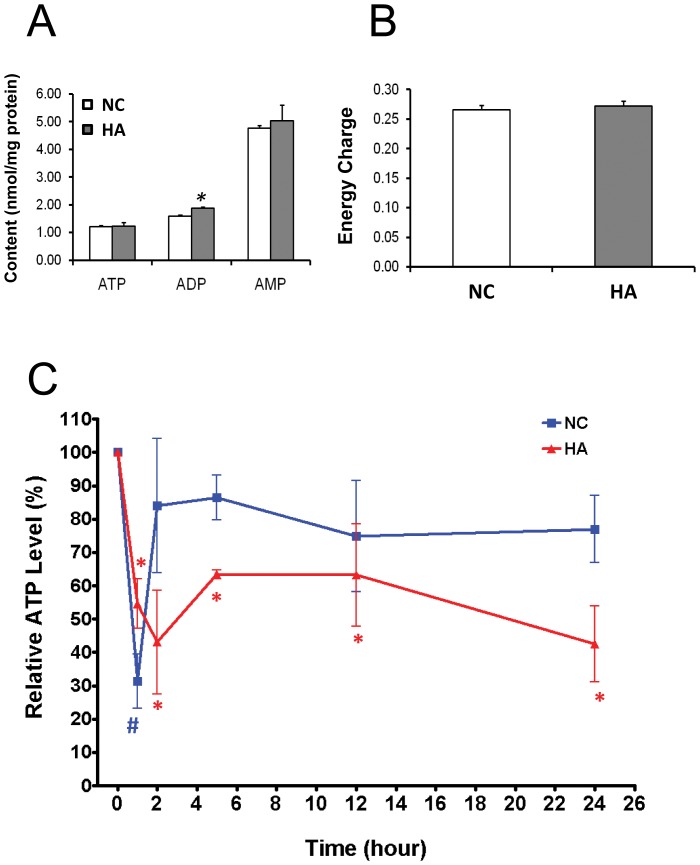
Equilibrium adenylate contents and hypoxia-induced changes in ATP level in the thoracic muscle of NC and HA flies. The equilibrium level of adenylates (i.e., ATP, ADP and AMP) in thoracic muscle was determined by HPLC. (A) No significant differences in ATP and AMP levels were determined between the HA flies living at 4 kPa O_2_ environment and NC controls living under room air condition (21 kPa O_2_) (n = 3; p>0.05). However, a slight but significant difference in ADP content was found in the HA flies (n = 3; * p<0.05). (B) EC showed no difference between HA and NC flies (n = 3; p>0.05). (C) Dynamic changes in ATP level under hypoxia in HA and NC thoracic muscle. A time course of changes in ATP level was measured in the thoracic muscle of HA and NC flies under hypoxic condition containing 2 kPa O_2_ during 24 hours. NC flies exhibited a transient decline of ATP within the first 2 hours of exposure. This decline was recovered following continued exposure (blue). HA flies also showed a decline of ATP level in the first 2 hours and stayed at this lowered level in the following period of hypoxia exposure time (red) (n = 6; **^#^** and * p<0.05).

The equilibrium level of adenylate (i.e., ATP, ADP and AMP) in thoracic muscle was determined by HPLC. Besides a slight but significant difference in ADP content, ATP, AMP and energy charge (EC) showed no difference between HA and NC flies demonstrating that energy homeostasis is identical in the control NC flies living at room air condition and HA flies living at 4 kPa O_2_ hypoxic condition (Figure 4A and 4B). However, when exposed to a further lowered oxygen level (i.e., 2 kPa O_2_), NC flies exhibited a transient decline of ATP within the first 2 hours of exposure. This decline was recovered following continued exposure (Figure 4C). In contrast, HA flies showed a decline of ATP level in the first 2 hours and stayed at this lowered level in the following period of hypoxia exposure, suggesting that a new ATP homeostasis was created in the HA flies in this new oxygen environment (Figure 4C). Using electron microscopy, we measured the mitochondrial volume density of NC flies placed in 2 kPa O_2_ for 2 and 6 hours and compared with room-air NC flies and found no difference, consistent with the ATP measurements (MitoVolDensity 0 hrs: 31±2%, n = 9; MitoVolDensity 2 hrs: 29±1%, n = 11; MitoVolDensity 6 hrs: 32±1%, n = 8; mean±SEM).

To address whether the drop in ATP levels at the earliest time point (1 hour) shown in Figure 4 could be explained by a structural phenotype, we examined electron micrographs and quantified observed structural modifications (Figure S1). We found that the NC and HA flies exposed to hypoxia for 1 hour did not have the mitochondrial honeycomb-like pattern nor a change in mitochondrial volume density, but instead showed subregions of deformed and swollen cristae within a subset of mitochondria (29±3% for NC, 14±3% for HA; mean ± SEM), consistent with the lowered levels of ATP at 1 hour. To separate whether the observed structural phenotype was influenced more by the genotype or by the environment, we interrogated whether that HA flies transferred to room oxygen level resemble the NC flies at this level. As with the NC and HA flies exposed to hypoxia for 1 hour, the HA flies raised in room air for one and two generations did not show a honeycomb-like pattern. We found that the mitochondrial volume density was significantly higher with the HA flies at room air for the first generation compared to the NC flies (38% HA vs. 30% NC; p<0.01) (Figure S1). However, by the second generation, there was no statistical difference (34% HA vs. 30% NC; p = 0.13). Moreover, the percent mitochondria with deformed cristae dropped from 12% (first generation) to 4% (second generation) compared to a baseline incidence of 5% for the NC. This suggests a quick reversion of the HA to the NC phenotype.

### Negative Geotaxis Behavior of HA and NC Flies in Room Air and Hypoxic Condition

The geotaxis behavior of HA and NC flies in a test for climbing ability was investigated by recording the time taken for half of a group of 10 male flies to climb the 5 cm wall of a glass tube under room air or hypoxic conditions; each group of flies was tested three times in succession. The mean journey time for both hypoxia-selected and control flies was <2 seconds under room air condition with no significant differences. In contrast, in the hypoxic environment, the climbing time was significantly increased in the hypoxia-selected flies to 16.4±1.3 seconds (mean ± SEM) (Table 1). However, the geotaxis behavior was totally abolished in the NC control flies, which showed little upward movement within 10 minutes after tapped to the bottom. Furthermore, the total locomotion activity was dramatically reduced in the NC flies. Most of the NC flies ceased movement even with physical disturbances of the tube by shaking or tapping. In contrast, the HA flies exhibited normal regular motion activities during the time exposed to the hypoxic environment (see Movie S3, S4, S5, S6).

**Table 1:** Medium journey time for hypoxia-selected and control flies to climb 2.5 cm distance in room air and 2 kPa O_2_ hypoxic environment.

	Room Air (second)	2 kPa O_2_ Hypoxia (second)
**Normoxia control flies (n = 90)**	**<2**	**>600**
**Hypoxia-selected flies (n = 90)**	**<2**	**16.4±1.3**

### Increased Sensitivity to Oxidative Stress in HA Flies

**Figure 5:**
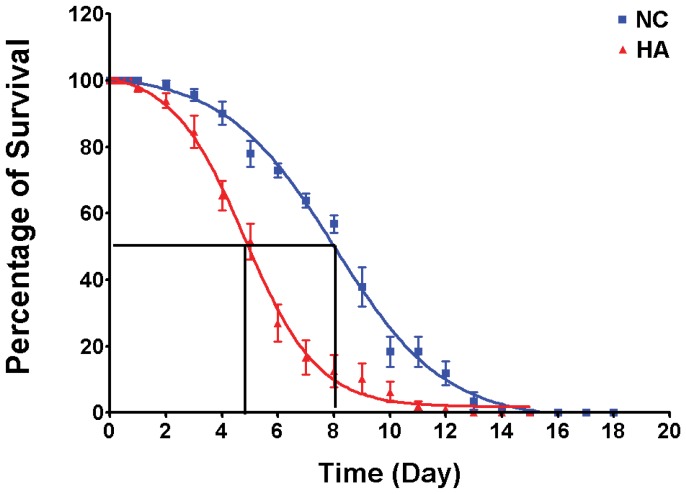
Increased sensitivity to oxidative stress in HA flies. A significantly shortened lifespan was determined in the HA flies (red) as compared to NC controls (blue) with 5 mM paraquat treatment under room air condition. The 50% survival of HA was 4.9±1.1 days, and the 50% survival of NC controls was 9.7±3.0 days (summarized from 6 separate experiments, p<0.01).

As mitochondria regulate not only energy metabolism through oxidative phosphorylation but also oxidative injury through ROS generation, the mitochondrial structural rearrangement observed in the hypoxia-selected flies suggests that these flies might have different ROS generation rate and, therefore lifespan when treated with ROS generation reagents. To test this hypothesis, we determined the lifespan of hypoxia-selected and control flies with paraquat treatment. Paraquat is a widely used superoxide generator that stimulates oxidative stress when catalyzed in mitochondria [27], [28]. We found that under the same treatment condition, the HA flies (both male and female) were more sensitivity to paraquat with a significantly shortened lifespan than controls as measured by a 50% survival rate. As shown in Figure 5, with 5 mM paraquat treatment, the 50% survival rate of male HA flies was significantly shortened (4.9±1.1 days) as compared to that of NC controls (9.7±3.0 days) (p<0.01). Similar results were obtained for female flies (data not shown). The 50% survival rate was also shorter in HA than NC when treated with 10 mM paraquat (data not shown).

It has been shown that a hypoxic environment may affect the food preference and intake of flies [29]. To determine the influence of food intake on paraquat treatment in the NC and HA flies, we measured food intake using a capillary feeding assay under room air condition, the same condition under which the paraquat treatment was performed. We found that the food intake was similar between HA (0.54±0.02 µl/mg body weight/day) and NC flies (0.49±0.02 µl/mg body weight/day) (mean ± SEM; n = 9, p>0.05), suggesting that NC and HA flies received a comparable dosage of paraquat during the treatment.

## Discussion

Muscle is an oxidative tissue of the body in which the production of cellular energy, in the form of adenosine triphosphate (ATP), occurs primarily via the process of oxidative phosphorylation in mitochondria. In order to sustain normal cellular function, mitochondria require a constant supply of fuels and oxygen. When oxygen delivery is impaired through hypoxia (such as decreased oxygen carrying capacity or decreased convective transport or in healthy individuals at altitude), a process of adaptation must occur to maintain cellular energy homeostasis. Indeed, it has been proposed that hypoxia is a key inducer of adaptation in skeletal muscle at altitude and with physical training [30], [31]. However, mechanisms underlying mitochondrial adaptation in a hypoxic environment are still largely uncertain. In this study, using a hypoxia-adapted *Drosophila melanogaster* line [23], we investigated the effects of CH on muscle and mitochondrial structure, muscle function, ATP metabolism and response to oxidative stress.

In the HA flies, we observed a reduction of myofiber size (i.e., 36%, p<0.01) and a slight, but significant, increase in mitochondrial volume density (i.e., 7%, p<0.01) (Figure 1). Interestingly, the reduction in muscle fiber size has also been observed in humans and animals that were exposed to chronic hypoxia including permanent high altitude residents and lowlanders or rodents that were exposed to hypoxia for a prolonged period of time [32], [33], suggesting that the mechanisms underlying CH-induced changes in muscle is conserved between vertebrates and invertebrates. It is believed that, on one hand, the reduced fiber size demonstrated a down-regulation of protein synthesis, which could significantly reduce energy demand. And, on the other hand, the reduced fiber size could potentially improve the efficiency of oxygen supply through vascular circulation in mammals or tracheal delivery in fruit fly. In terms of mitochondrial volume density, loss of skeletal muscle mitochondrial density is a consistent finding in humans at altitude and patients with respiratory disease [34], [35]. For example, a reduction in mitochondrial volume density was reported in CH exposed human subjects including individuals from the Tibetan population [35], [36]. Such reduction in mitochondrial volume density was retained even in the second-generation who were born and raised at a much lowered altitude [32], suggesting that the reduced mitochondrial volume density is a heritable trait in this high altitude adapted Tibetan population. However, a muscle-type dependent, CH-induced change in mitochondrial volume density has been found in rats treated with CH where the mitochondrial volume density was increased in the soleus muscle but decreased in the extensor digitorum longus muscle in the same animal treated with CH [33]. The slight, but significant, increase in mitochondrial volume density detected in the HA flies suggested that change in mitochondrial volume density might be an adaptive mechanism that potentially maximizes the efficiency of aerobic respiration under oxygen limited conditions.

Electron microscope tomography allowed us to determine detailed 3D mitochondrial structural modifications in the HA flies. In the thoracic muscle of HA flies, we found localized cristae fragmentation and matrix swelling (Figure 2), but to a lesser extent than reported in other studies where mitochondria enlargement, cristae rupture and loss were commonly described [37], [38], [39], [40]. Instead of unrepaired cristae rupture, we found that the ends of the fragmented cristae membranes had rejoined (annealed) that appeared to be a repair mechanism. This annealing produced cristae with larger widths. These wider cristae may be a natural consequence of local changes in osmolarity because of mixing of the intracristal and matrix compartments upon rupture and subsequent annealing, consistent with studies of cristae width changes influenced by osmolarity [41]. The wider cristae may also reflect an energy minimum of the bilayer membrane based on a thermodynamic model of curvature [42], meaning that the combination of the shorter cristae and the curvature of their ends produces wider cristae to satisfy an energy minimum.

Cristae fragmentation and matrix swelling have been reported through a variety of perturbations [43], [44], [45], [46] including myocardial ischemia [47], [48]. The cristae fragmentation observed in our longitudinal sections produced an interesting honeycomb-like pattern in cross-section that was not observed in NC mitochondria (Figure 3). Interestingly, similar honeycomb-like cristae structures have been reported in various previous studies. For example, it has been observed in a *Drosophila* line carrying pathogenic ATP6 mutation, which showed a honeycomb appearance consisting of numerous cristae vesicles that were often interconnected with each other [49]. It is noteworthy that this group also reported fenestrated cristae in the mutant samples that we found to be a hallmark of HA mitochondria. In addition, in Barth syndrome patients [50] and a mouse model of the disease [51], a honeycomb structure was also observed inside mitochondria. Another similar pattern was reported in TIM44-transfected COS7 cells [52]. In all of these instances, however, the honeycomb patterns were different than what we observed and appeared to be paracrystalline in the mitochondria described by Acehan and coworkers and Wada and Kanwar. These authors noted that this pattern could conceivably be derived from mitochondria under stress conditions, including early stages of ischemia. It seems natural to us to also suggest that the cristae fragmentation, fenestration, and the novel honeycomb-like pattern observed in HA mitochondria is due to the stress of hypoxia. However, we cannot exclude the contribution of genetic variation in the HA flies derived from hypoxia selection. Compensation for hypoxic stress might be manifested by the increase in cristae membrane abundance and thus ATP-producing capacity. The increased cristae fragmentation measured in HA mitochondria is consistent with the report that oxygen deficiency increased the cristae content of mitochondria in myocardial cells [53]. Taken altogether, these findings, including ours, demonstrate that morphological plasticity contributes to mitochondrial adaptation under hypoxic environmental constraint.

Maintaining an effective ATP level is essential for muscle function. Since both cross-bridge cycling at the sarcomere during contraction and calcium reuptake to the sarcoplasmic reticulum during relaxation are heavily dependent on ATP hydrolysis, a compromise in cellular energy can lead to more rapid fatigue in exercising muscle. For example, in cardiac muscle, energetic impairment has been associated with the pathogenesis of hypertrophic cardiomyopathy and sudden cardiac death [54]. We detected similar levels of adenylates and energy charge between HA (living in 4 kPa O_2_ severe hypoxic environment) and NC (living at 21 kPa O_2_ room air condition) with a slightly higher level of ADP in the muscle samples of HA flies (Figure 4A-B), demonstrating that the adenylate homeostasis is maintained well in this hypoxia-adapted *Drosophila* lines at their “native” hypoxic environment. As a previous metabolomic study in the same *Drosophila* line did not find evidence supporting a significant up-regulation of anaerobic metabolic activities in the HA flies [26], such well-maintained adenylate homeostasis is very likely through aerobic metabolism in well-functioning mitochondria. Indeed, the same study suggested that HA flies produced more ATP per glucose and created fewer protons than control flies. And HA flies may have lower rates of glycolysis, less acidosis, and more efficient use of substrate during acute hypoxic stress [26]. This notion is further supported by the observation that, under hypoxic condition (i.e., 2 kPa O_2_), HA flies showed normal, even though delayed, geotaxis behavior. However, in NC flies, such geotaxis activity was totally abolished (Table 1), suggesting that the HA muscle was well functioning in the severe hypoxic environment.

The dynamic change in ATP level following acute hypoxia treatment (i.e., 2 kPa O_2_) was measured during a 24 hours exposure time. The NC flies showed a rapid drop in the first hour and an almost full recovery at the 2 hour time point in this hypoxic environment. In contrast, the HA flies exhibited a slower drop in the first 2 hours followed by a partial recovery and remained at a lowered ATP level (∼60% of the original level) for the rest of the experimental time (Figure 4C). Such a lowered ATP level might result from higher physical and cellular activities in HA flies as compared to NC controls under hypoxic condition. As mentioned above, the geotaxis activity was well retained in the HA flies as measured after 2 hours treatment with 2 kPa O_2_. In contrast, following the same treatment time, even though the ATP level was recovered to normal value, the majority of NC flies remained motionless with totally abolished geotaxis activities in this hypoxic environment (Table 1), which may also indicate that the efficiency of muscle energetic regulation is different between HA and NC flies. Indeed, it has been reported that chronic hypoxia improves energy supply regulation in the cardiac muscle of mice [55]. As shown in Figure 2, HA flies generated fragmented cristae and a larger cristae surface area in the mitochondria during development in their “native” chronic hypoxic environment. Such increased cristae fragmentation and cristae abundance in the HA mitochondria may play a critical role in increasing energy supply by providing an enlarged reaction surface and efficient proton flow under low oxygen condition. Consequently, this adaptive structural modification preserved the muscle function and the physical activity of HA flies in hypoxia.

It has been found that CH induces oxidative stress in muscle. For example, climbers who have been exposed for prolonged periods to extreme altitude exhibit evidence of oxidative stress in skeletal muscles [56], [57]. Similar oxidative changes in muscle biopsies have also been observed in hypoxic chronic obstructive pulmonary disease (COPD) patients but not in obstructed COPD patients who do not exhibit hypoxia [58]. Although the mechanisms for hypoxia-induced ROS formation in skeletal muscle, or in any other tissue, are still not fully understood, it has been proposed that the mitochondrion is the strongest candidate for the source of hypoxia-induced ROS formation [59], [60], [61]. Using paraquat (1,1′-dimethyl-4,4′-bipyridinium dichloride), a ROS generator through mitochondria metabolism, we compared the susceptibility to oxidative stress between HA and NC flies. We found that HA flies are more vulnerable to paraquat treatment with a significantly shortened lifespan than NC when treated with the same dosage (Figure 5). Paraquat has been widely used to stimulate superoxide production in organisms and cells. Previous studies have shown that superoxide production by paraquat occurs in the mitochondrial matrix where it was reduced by complex I to form the paraquat radical cation that then reacted with oxygen to form superoxide [27], [28]. Such superoxide production can cause extensive mitochondrial oxidative damage [62], [63], [64], [65]. Our previous studies have found that the activity of respiratory complex I was elevated in HA flies [26], [66]. An increased complex I activity may increase the level of paraquat-derived ROS production. Furthermore, it has been shown that the ROS level and its defense mechanisms were reduced in flies living in chronic constant hypoxic condition [66], [67]. Therefore, the increased sensitivity to paraquat treatment in HA flies might be a combined result from both an increased production of paraquat-derived ROS and a reduced ROS defense.

In summary, we observed significant ultrastructural modifications with increased cristae fragmentation and abundance in the mitochondria of thoracic muscle from hypoxia-adapted *Drosophila melanogaster*. Our results suggest that such morphological rearrangement may potentially be an important adaptive mechanism that plays a critical role in preserving adenylate homeostasis and metabolism as well as muscle function under chronic hypoxic conditions.

## Materials and Methods

### *Drosophila Melanogaster* Stocks and Culture

The parental *D. melanogaster* lines, HA and control flies used in this study were described previously. Briefly, 27 isogenic *Drosophila melanogaster* lines were balance-pooled to generate the parental population for experimental selection of hypoxia-adapted flies and controls. These 27 isogenic lines had different recovery times from acute anoxia stupor and eclosion rates under hypoxic conditions, demonstrating genetic diversity in terms of hypoxia response [23]. The hypoxia-selected (HA) and control (NC) flies were used for the current study. The flies were cultured at room temperature on standard cornmeal/agar medium. The HA flies were cultured under the hypoxic condition containing 4 kPa O_2_ balanced with nitrogen. And the NC flies were cultured under room air (21 kPa O_2_) condition.

### Negative Geotaxis Assay

The climbing test was performed according to a previous description with modifications [68]. A group of 10 male flies (5–7 days old) were placed in a 15-ml glass tube with food and were gently tapped to the bottom of the tube. The time taken for half of the knocked-down flies to climb 2.5 cm of the tube wall was recorded. Each group was tested 10 times in room air (21 kPa O_2_) or hypoxic condition (2 kPa O_2_) in a computer-controlled atmosphere chamber (Model 8301055; Coy laboratory Products Inc, Grass Lake, MI).

### Measurement of Adenylate Contents and ATP Homeostasis

Adult flies were rapidly frozen using liquid nitrogen. Thoracic muscle from 40∼50 flies was isolated on dry-ice and homogenized in 300 µl pre-chilled 2.3 M Perchloric acid (PCA) to extract free nucleotides. The homogenate was placed on ice for 15 min and then centrifuged at 13,000g for 5 min. 1 M K_2_CO_3_ was added to the supernatant until the pH was neutralized. After centrifugation, the supernatant was filtered with a 0.22 µm membrane and stored in a −20°C freezer until use for HPLC analysis [69].

The HPLC system (Shimadzu, Japan) consists of a Shimadzu CBM-20A controller, a SPD-20A variable wavelength UV detector, a LC-6AD pump and a Phenomenex C18 column (25 cm×4.6 cm; 5 µm, 100A). The nucleotides extracted from the thoracic muscle (100 µl) were run in a HPLC program to separate nucleotides (100% A solution (30 mM KH_2_PO_4_,pH 6.0) to 60% B solution (30 mM KH_2_PO_4_, pH 6.0, and 15%ACN) within 15 min, flow rate 1 ml/min), and the chromatographic peak at 254 nm was recorded. The standard solution containing certain concentrations of nucleotides was run in the same HPLC program. Different concentrations of nucleotides were run in the program and calibration curves of ATP (0.5–5.0 µM), ADP (0.5–5 µM) and AMP (4.0–15.4 µM) were generated.

Change in the ATP level following hypoxia treatment was measured by using the ATPLite Adenosine TriPhosphate (ATP) assay kit (PerkinElmer, Boston, MA) following the manufacturer’s instruction. Groups of 1-week old male NC or HA flies were collected (10 flies per group) and treated in a 2 kPa O_2_ hypoxic chamber. Flies were collected at different time points during treatment (0, 1, 2, 5, 12 and 24 hours). Three groups of flies from NC and HA were analyzed at each time point. The ATP level was normalized against protein level measured in each sample.

### Paraquat Treatment and Lifespan

Paraquat (1,1′-dimethyl-4,4′-bipyridinium dichloride) stock solution (100 mM) was diluted to 5 mM or 10 mM with 5% sucrose solution for treatment. 5–8 old flies were collected and kept in empty vials for 1 hour prior to treatment. Five vials containing 10 male flies each and 5 vials containing 10 females each were tested for each concentration per experiment. Survival of flies was counted every 24 hours. Freshly prepared PQ solution was added to the vials every 48 hours. The survival curve and 50% survival rate of each experiment was generated and determined using a sigmoidal nonlinear regression model (GraphPad Prism 4 software, San Diego, CA).

### Capillary Feeding Assay

Daily food intake of NC and HA flies was measured in 5–7 days old male flies using a capillary feeding assay as previously described [29]. Briefly, 2 pieces of Whatman filter wetted with 0.5 ml of distilled water was placed at the bottom of a *Drosophila* Culture vial (Genessee Scientific, San Diego, CA) to provide a water source and moist condition that prevent evaporation of feeding solutions. Flies in groups of 10 were added to the vials and the tube was sealed with Bonded Dense Weave Cellulose Acetate flug which provides an excellent barrier to mite infestation and reduces evaporation (Genessee Scientific, San Diego, CA). A vertical cut on the flug was introduced to hold the capillary feeder. Food was provided by a 75 µl micro capillary tube (Warner instrument Corp., Hamden, CT). Capillaries were filled with 25 µl of liquid diet (5% sucrose or apple juice). Fresh food was provided every 24 hours. The height of the liquid column in the capillaries was measured at each food change and for 72 hours. Control experiments were performed to measure the rate of water evaporation under experimental condition using tubes without flies.

### Stereological Measurement of Mitochondrial and Myofiber Volume Fractions

For mitochondrial volume density measurements, eleven TEM images were analyzed for the NC and 15 TEM images for the HA as follows. A 9×13 rectangular grid (chosen for ease of use with Photoshop) was overlaid on each image, and mitochondria and cytoplasm lying under intercepts were counted. The relative volume density (volume fraction) of mitochondria was expressed as the ratio of intercepts coinciding with this organelle relative to the intercepts coinciding with cytoplasm+mitochondria (including the myofibers) and reported as a percentage. Mitochondrial and myofiber numbers were measured from 6 NC and 10 HA images and normalized to the cross-sectional areas of the cells in each image measured with ImageJ (National Institutes of Health). The cross-sectional area of 69 myofiber bundles was measured from the TEM images.

### Electron Microscopy and Tomography

For electron tomography, sections were cut from the same blocks used for conventional transmission electron microscopy at a nominal thickness of 0.5 µm and collected on 100∶100 clamshell grids. These sections were poststained for 15 min in a 2% uranyl acetate solution followed by 15 min in Sato lead solution. Two sizes of colloidal gold particles, 15 and 20 nm in diameter, were deposited on opposite sides of the section to serve as fiducial cues. For stability in the beam, the section was coated with carbon. For each reconstruction, a single series of images was collected with a JEOL 4000EX intermediate high voltage electron microscope operated at 400 kV. The specimens were irradiated for about 30 min before initiating a tilt series to limit anisotropic specimen thinning during image collection. During data collection, the illumination was held to near parallel beam conditions. Single-tilt series were recorded using a 4 k×4 k slow scan CCD camera controlled by the program serialEM at 12,000 magnification. Angular increments of 2° usually from −60° to +60° about an axis perpendicular to the optical axis of the microscope were achieved using a computer-controlled goniometer to increment accurately the angular steps. The pixel resolution was 1.2 nm. To improve the signal-to-noise ratio, each image was binned down two times by averaging adjacent pixels so that the final pixel resolution was 2.4 nm. The IMOD software package [70] was used for the complete image processing unless there were problems, and then TxBR software was used to refine the alignment and reconstruction [71]. Five NC and five HA mitochondrial volumes were generated. Volume segmentation was performed by manual tracing in the planes of highest resolution (*X-Y*) with the program Xvoxtrace [72]. The reconstructions were visualized using Analyze (Mayo Foundation) and the surface-rendering graphics of Synu (National Center for Microscopy and Imaging Research) as described by Perkins *et al.* [72]. These programs allow one to step through slices of the reconstruction in any orientation and to model and display features of interest in three dimensions.

Measurements of mitochondrial outer and crista membrane surface areas and the number of cristae per mitochondrion were made within the 10 segmented tomographic volumes by the program Synuarea (National Center for Microscopy and Imaging Research, University of California San Diego). For the cristae width measurements, all the mitochondria generated for our 3D analysis were used and a total of 100 cristae per condition (HA long cristae, HA fragmented cristae, and NC cristae) were measured with ImageJ. The cristae membrane was included in the width.

### Statistical Analysis

All values were represented as means ± SEM. Statistical significance was calculated by ANOVA analysis. Differences were considered significant if p<0.05.

## Supporting Information

Figure S1 **Mitochondrial structural analysis for (1) NC flies exposed to 2% O_2_ for 1 hour, (2) HA flies exposed to 2% O_2_ for 1 hour, (3) HA flies raised in room air for 1 generation, and (4) HA flies raised in room air for 2 generations.** (A and B) Example mitochondria of the two structural phenotypes observed. (A) Region inside a NC flight muscle mitochondrion with deformed and swollen cristae (*) observed when NC or HA flies are exposed to 2% O_2_ for 1 hour. Whereas commonly observed, this cristae deformation is still the minority; more mitochondria appear like (B) Typical mitochondrion showing the normal morphology of well-ordered and densely packed cristae in NC or HA flight muscle. (C) Typical mitochondrion from HA flies raised in room air for one generation, showing normal morphology with densely packed cristae. (D) Typical mitochondrion from HA flies raised in room air for two generations, again showing normal morphology. Scale  = 500 nm applies to all panels. (E) Mitochondrial volume density. The mitochondrial volume density was significantly higher with the HA flies at room air for the first generation compared to the NC flies (38% HA room air, 1 gen. vs. 29% NC room air-taken from Figure 1; p<0.01). However, by the second generation, there was no statistical difference (34% HA room air, 2 gen. vs. 29% NC room air; p = 0.13). There was no statistical difference between HA and NC flies cultured in 2% O_2_ for 1 hr (p = 0.38) or compared with HA 4% O_2_– taken from Figure 1 (p = 0.17) or NC room air (p = 0.12), respectively. n = 10 for all samples. (F) Percentage of mitochondria with subregions of deformed and swollen cristae, as shown in (A). The NC flies cultured in 2% O_2_ for 1 hr had significantly more deformed cristae than did the HA flies cultured in 2% O_2_ for 1 hr (** p<0.001). Interestingly, the HA flies cultured in room air for 2 generations had less deformed cristae compared with the HA flies cultured in room air for 1 generation (* p<0.05) and was comparable to the NC room air. n = 10 for all samples. Not surprisingly, the HA flies grown in 4% O_2_ had significantly more deformed cristae than the NC room air cristae.
(TIF)

Movie S1 **A mitochondrion from NC fly thoracic muscle.** Movie shows the three-dimensional details of a medium-sized mitochondrion reconstructed using electron tomography. *Clip1*: A rapid sequence through 105 2.4 nm-thick slices of the tomographic volume. *Clip2*: Rotations of the surface-rendered volume after segmentation of the inner and outer membranes. The outer membrane is translucent to visualize the cristae. 42 cristae are present. *Clip3*: Rotation showing only 8 representative cristae after removal of the inner boundary membrane.
(MP4)

Movie S2 **A mitochondrion from HA fly thoracic muscle.** Movie shows the three-dimensional details of a large mitochondrion reconstructed using electron tomography. *Clip1:* A rapid sequence through 50 4.8 nm-thick slices of the tomographic volume. *Clip2*: Rotations of the surface-rendered volume after segmentation of the inner and outer membranes. The outer membrane is translucent to visualize the cristae. 641 cristae are present, many of which are branched. *Clip3*: Rotation showing only 11 representative cristae that demonstrate their fragmentation. *Clip4*: Zoom in and rotation showing 4 adjacent cristae. *Clip5:* Restoration of all the cristae and partial rotation ending with the slice through the left-hand portion of the surface rendered mitochondrion.
(MP4)

Movie S3 **Geotaxis activity of NC flies under room air condition.**
(WMV)

Movie S4 **Geotaxis activity of HA flies under room air condition.**
(WMV)

Movie S5 **Geotaxis activity of NC flies under hypoxic condition with 2** **kPa O_2_.**
(WMV)

Movie S6 **Geotaxis activity of HA flies under hypoxic condition with 2** **kPa O_2_.**
(WMV)
