# Supplementary figures and images for: Ultrastructural Modifications in the Mitochondria of Hypoxia-Adapted Drosophila melanogaster
Source: PLoS One. 2012 Sep 19;7(9):e45344. doi: 10.1371/journal.pone.0045344 (PMC3446896; doi:10.1371/journal.pone.0045344)

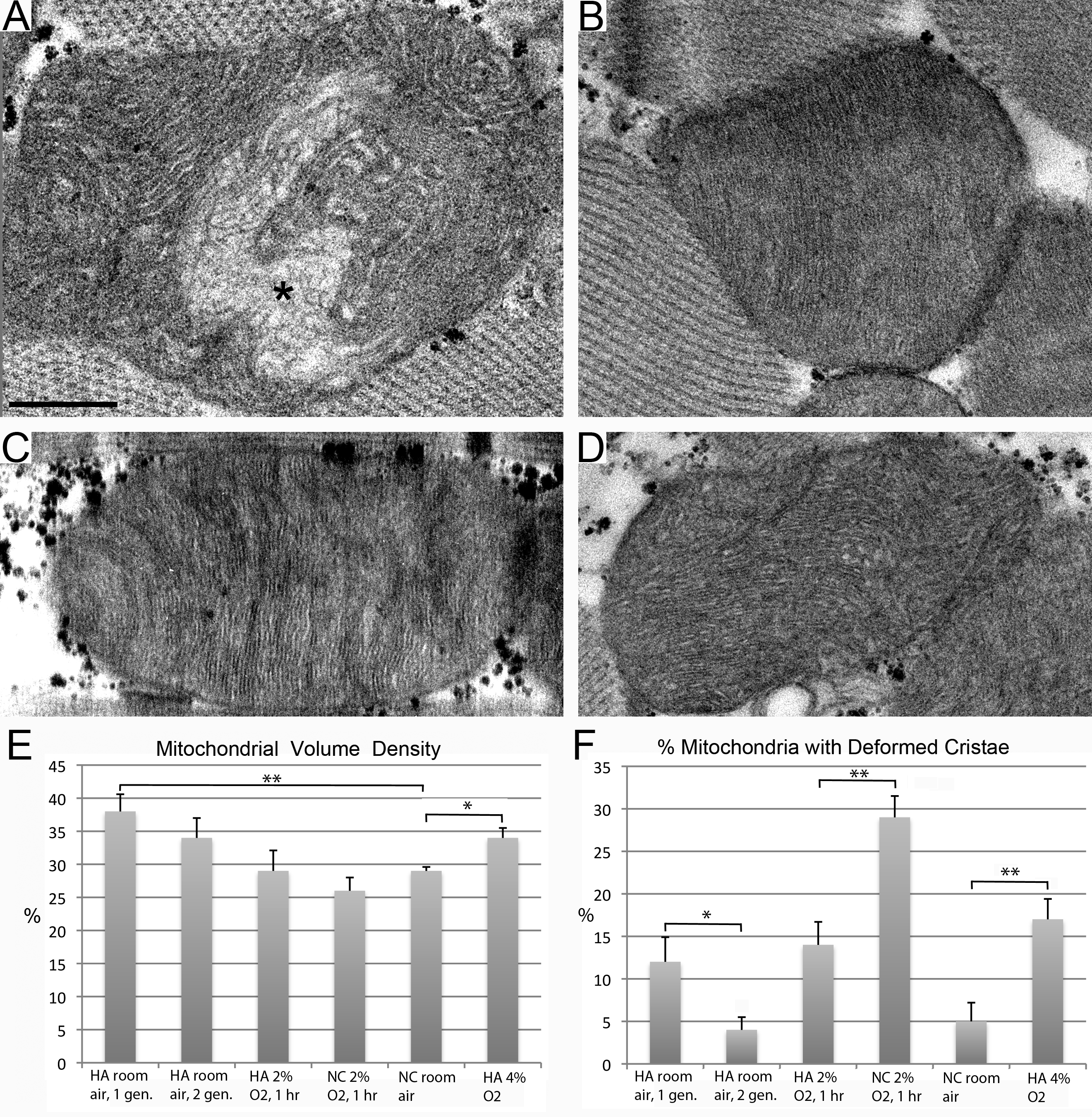

Supplement: Figure S1 — Mitochondrial structural analysis for (1) NC flies exposed to 2% O2 for 1 hour, (2) HA flies exposed to 2% O2 for 1 hour, (3) HA flies raised in room air for 1 generation, and (4) HA flies raised in room air for 2 generations. (A and B) Example mitochondria of the two structural phenotypes observed. (A) Region inside a NC flight muscle mitochondrion with deformed and swollen cristae (*) observed when NC or HA flies are exposed to 2% O2 for 1 hour. Whereas commonly observed, this cristae deformation is still the minority; more mitochondria appear like (B) Typical mitochondrion showing the normal morphology of well-ordered and densely packed cristae in NC or HA flight muscle. (C) Typical mitochondrion from HA flies raised in room air for one generation, showing normal morphology with densely packed cristae. (D) Typical mitochondrion from HA flies raised in room air for two generations, again showing normal morphology. Scale = 500 nm applies to all panels. (E) Mitochondrial volume density. The mitochondrial volume density was significantly higher with the HA flies at room air for the first generation compared to the NC flies (38% HA room air, 1 gen. vs. 29% NC room air-taken from Figure 1; p<0.01). However, by the second generation, there was no statistical difference (34% HA room air, 2 gen. vs. 29% NC room air; p = 0.13). There was no statistical difference between HA and NC flies cultured in 2% O2 for 1 hr (p = 0.38) or compared with HA 4% O2– taken from Figure 1 (p = 0.17) or NC room air (p = 0.12), respectively. n = 10 for all samples. (F) Percentage of mitochondria with subregions of deformed and swollen cristae, as shown in (A). The NC flies cultured in 2% O2 for 1 hr had significantly more deformed cristae than did the HA flies cultured in 2% O2 for 1 hr (** p<0.001). Interestingly, the HA flies cultured in room air for 2 generations had less deformed cristae compared with the HA flies cultured in room air for 1 generation (* p<0.05) and was comparable to the N [file pone.0045344.s001.tif]
